# Estimation of perioperative invasiveness of colorectal endoscopic submucosal dissection evaluated by energy metabolism

**DOI:** 10.3164/jcbn.18-12

**Published:** 2018-04-11

**Authors:** Daisuke Chinda, Tadashi Shimoyama, Kuniaki Miyazawa, Tetsu Arai, Shiro Hayamizu, Miyuki Yanagimachi, Toshiaki Tsukamoto, Kazuki Akitaya, Tetsuya Tatsuta, Shogo Kawaguchi, Hidezumi Kikuchi, Hiroto Hiraga, Manabu Sawaya, Hirotake Sakuraba, Tatsuya Mikami, Shinsaku Fukuda

**Affiliations:** 1 Department of Gastroenterology and Hematology, Hirosaki University Graduate School of Medicine, 5 Zaifu-cho, Hirosaki, Aomori 036-8562, Japan; 2 Department of Endocrinology and Metabolism, Hirosaki University Graduate School of Medicine, 5 Zaifu-cho, Hirosaki, Aomori 036-8562, Japan; 3 Department of Rehabilitation, Hirosaki University Hospital, 5 Zaifu-cho, Hirosaki, Aomori 036-8562, Japan; 4 Division of Endoscopy, Hirosaki University Hospital, 5 Zaifu-cho, Hirosaki, Aomori 036-8562, Japan

**Keywords:** endoscopic submucosal dissection, energy metabolism, early colorectal cancer, perioperative period, indirect calorimeter

## Abstract

The aim of this study was to assess the perioperative invasiveness of endoscopic submucosal dissection for colorectal cancer quantitatively by using energy metabolism. In fifty-three patients who underwent endoscopic submucosal dissection for colorectal cancer, resting energy expenditure using an indirect calorimeter, body weight and basal energy expenditure using the Harris–Benedict equation before and after endoscopic submucosal dissection. Resting energy expenditure/body weight and resting energy expenditure/basal energy expenditure were 19.7 ± 2.5 kcal/kg/day and 0.96 ± 0.12 on the day of endoscopic submucosal dissection, whereas one day after the endoscopic submucosal dissection they increased to 21.0 ± 2.9 kcal/kg/day and 1.00 ± 0.13 (*p*<0.001 and *p*<0.05, respectively). The stress factor on the postoperative day 1 was computed as 1.06. The increase was lower comparing with that experienced for surgery, suggesting that the perioperative invasiveness of colorectal endoscopic submucosal dissection is lower in comparison to that during surgery. Furthermore, in spite of technical difficulty, stress factor of colorectal endoscopic submucosal dissection was approximately equal to that of gastric endoscopic submucosal dissection. (The study of the resting energy metabolism and stress factor using an indirect calorimeter in the perioperative period of endoscopic operation: UMIN000027135)

## Introduction

In Japan, endoscopic submucosal dissection (ESD) for early gastric and esophageal cancer has been approved by health insurance system in 2006 and 2008, respectively. In comparison with these procedures, colorectal ESD has been associated with several difficulties, including need for higher level of technical skills and the risk of severe peritonitis due to intestinal perforation. Therefore, ESD for early colorectal cancer was initially approved as an advanced medical technology in July 2009, and finally approved by health insurance system in April 2012. At present, many reports have shown that colorectal ESD is a highly safe and useful procedure.^(1–5)^

On the other hand, it is known that in the perioperative period of surgery, metabolism varies greatly due to invasion, and the increase of stress modulates the energy requirements.^(6–12)^ Both resting energy expenditure (REE) per kg of body weight (BW) and stress factor have been used as quantitative markers of invasiveness during the perioperative period. Our previous study indicated that both REE/BW and stress factor increased during the perioperative period of ESD for early gastric cancer while the increase was lower in comparison with those experienced for surgery.^(13)^

For colorectal cancers, increase of calorie consumption and stress factor have been shown in surgery. However, in ESD for colorectal cancers, no study has examined these factors and invasiveness of ESD has never been compared with surgery. Although the invasiveness of ESD was smaller than surgery for gastric cancers, there are considerable differences between colorectal and gastric ESD, including not only technical difficulties but also perioperative management. In gastric ESD, patients only fast after dinner on the day before ESD and undergo ESD using pethidine hydrochloride and midazolam or diazepam. On the other hand, colorectal ESD requires bowel cleansing before the procedure. Patients fast from morning of the day before ESD and receive drip infusion, and administer whole bowel irrigation in the evening before and on the day of ESD. Colorectal ESD is performed in the awakening state only with analgesia by pethidine hydrochloride because patients may need to change the posture to make the ESD procedure easier. Therefore, it is possible that the invasiveness of colorectal ESD is different from that of gastric ESD.

The present study was undertaken in order to assess the invasiveness in the perioperative period of ESD for colorectal cancer by evaluating the change of REE/BW and stress factor. These quantitative indicators could be useful to explain the invasiveness of ESD to the patients with colorectal tumors. In addition, REE/BW and stress factor of colorectal ESD were compared with those of surgery or gastric ESD written in previous papers.

## Materials

**Fig. 1:**
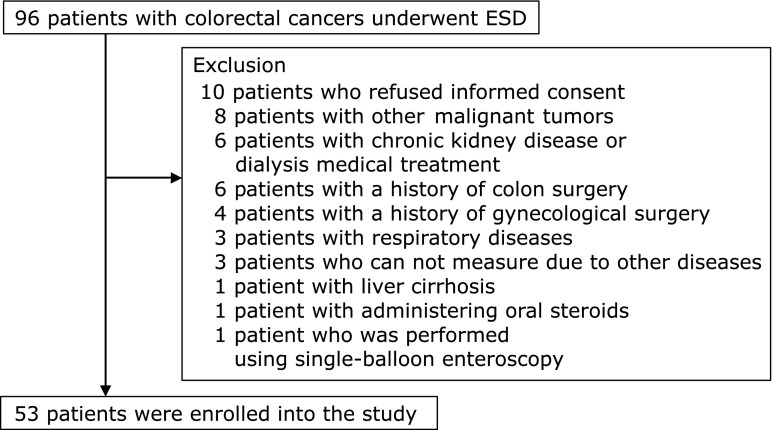
Flowchart of this study.

Patients who underwent colorectal ESD in Hirosaki University Hospital during July to December in 2013 and January to November in 2016 were prospectively enrolled into the study. The period from 2014 to 2015 was interrupted due to repair of the indirect calorimeter. We excluded patients who had disorders which affect the measurement of indirect energy requirements as shown in Fig. 1.^(14–16)^ Patients were also excluded if they had difficulty to insert the colonoscope (due to gynecological surgery, etc.) or to collect breath samples due to other diseases (cleft lip, atlantoaxial dislocation, etc.). ESD was performed using a conventional single channel endoscope (PCF-Q260JI or GIF-Q260J; Olympus, Tokyo, Japan) with hood. ESD procedure was mainly performed using Hook Knife (KD-620LR, Olympus). We also used a high frequency generator with an automatically controlled system, ICC200 and VIO300D thereafter (both supplied by ERBE, Tübingen, Germany). In 32 patients (60.4%), pethidine hydrochloride (25–50 mg/body) was used for sedation. All ESD procedures were performed by Board Certified Endoscopists of The Japan Gastroenterological Endoscopy Society.

Measurement of REE was performed in the same way as shown in our previous study.^(13)^ In brief, REE was measured using indirect calorimeter METAVINE-N VMB-002N (VINE, Tokyo, Japan). METAVINE-N computes REE using the oxygen concentration and respiration rate in the breath; it does not use the carbon dioxide concentration.^(7,17–21)^ Since respiratory quotient remains approximately constant at the time of rest, and the inter-individual differences are also considered to be small, REE can be measured assuming a constant respiratory quotient under bed-rest.

Each patient fasted for more than 20 h since morning on the day before ESD, and REE was measured after 30 min of bed-rest early in the morning on the day of ESD. Subsequently, ESD was conducted; upon completion and without any food intake, and on the next day, REE was measured once again early in the morning. REE was measured three times, and was computed as the mean when the variations were less than 100 kcal. When the variations were >100 kcal, a fourth measurement was performed and REE was computed as the mean from three measurements, excluding the one that was further from the mean of the two median values. Some patients were nervous at first and breathed in deep or shallow bringing unstable results. In other cases, results were not stable because of insufficient setting of the mask. Therefore, we increased the number of measurements to obtain stable results as possible. BW was also measured prior to ESD and within two days after ESD before stating oral ingestion. Subsequently, REE per kg of BW (REE/BW) between pre and post ESD procedure was compared.

Further, basal energy expenditure (BEE) was calculated using the Harris–Benedict equation based on the height and BW of each patient. According to Long’s method,^(22)^ REE can be determined by multiplying BEE with activity and stress factors. Stress factors are the markers of hyper-metabolic status.^(13,16–20,23)^ Since the preoperative and postoperative activity factors are the same in a state of rest, assuming the REE/BEE on the day of ESD to be 1, and the REE/BEE measured on the postoperative day 1 can be considered as the stress factor.

This study was approved by the Hirosaki University ethics committee. Prior to the admission or on the day before ESD procedure, the details of the procedure and the research objective were explained to the participants and written consent was obtained from all participants who were willing to collaborate.

In the analysis, continuous variables were expressed as mean ± SD. Paired *t* test was used for comparison of REE, BW, REE/BW, and stress factors between preoperative and postoperative ESD states, and a *p* value less than 0.05 was considered statistically significant using SPSS ver. 24. OJ (SPSS Inc., Chicago, IL).

## Results

### Patients’ characteristics

During the study period, a total of 53 patients who underwent colorectal ESD were eligible to enter the study (Fig. 1). Sample size was calculated using significance level (α) of 0.05 and power (1 – β) of 0.80. The value of the standard deviation (δ) was calculated using the prediction value based on the data of the previous study of gastric ESD. Consequently, a sample size of 53 had statistical power of 0.985 (REE/BW) and 0.993 (stress factor), respectively.

Table 1 shows the basic characteristics of the patients. The operation period included the period of magnifying observation time prior to ESD procedure. The ellipse with the length and breadth of the resection specimen was used to express approximate resection area. For 14 patients with multiple lesions, the total resection area of all the lesions was computed. Complications of ESD were observed in 5 patients, including perforation in one case, postoperative bleeding in two cases, and fever >38°C in one case. All the patients received successful medical treatment and no patient required additional surgery for complications.

### REE, BW and BEE

Table 2 shows the changes in REE, BW and BEE in the perioperative period of ESD. REE on the day of ESD and the next day were 1,107.0 ± 204.4 kcal and 1,139.9 ± 185.2 kcal, respectively. There was no significant difference between pre and postoperative REE. BW was a significant decrease from 56.5 ± 9.4 kg to 54.8 ± 8.9 kg after ESD (*p*<0.001). The BEE on the day of ESD was 1,075.8 ± 148.5 kcal, while it was elevated to 1,139.9 ± 141.5 kcal on the next day (*p*<0.001).

### REE/BW and REE/BEE

**Fig. 2:**
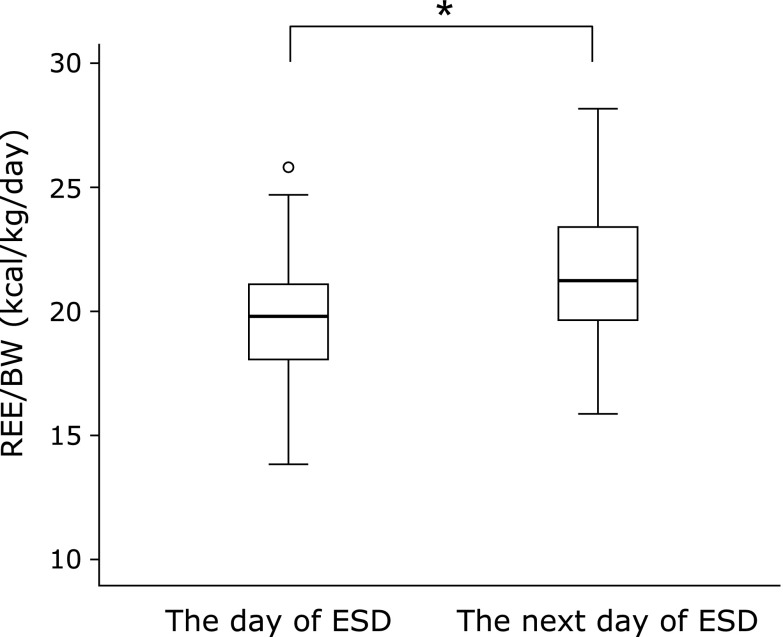
The change in resting energy expenditure/body weight (REE/BW) during the perioperative period of colorectal endoscopic submucosal dissection (ESD). Data are shown as mean ± SD. *p<0.001: compared with the value of the day of ESD.

**Fig. 3:**
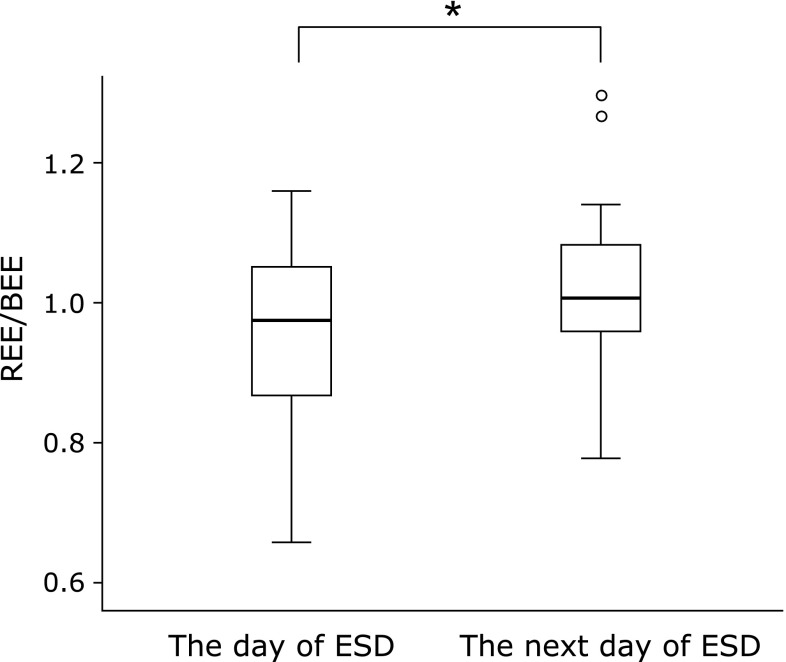
The change in energy expenditure/basal energy expenditure (REE/BEE) during the perioperative period of colorectal endoscopic submucosal dissection (ESD). Data are shown as mean ± SD. *p<0.05: compared with the value of the day of ESD.

REE/BW was increased in 43 out of 53 patients (81.1%). On the day of ESD, REE/BW was 19.7 ± 2.5 kcal/kg/day, and it was significantly increased to 21.0 ± 2.9 kcal/kg/day on the next day (*p*<0.001, Fig. 2). Similarly, increase of REE/BEE was seen in 42 patients (79.2%). REE/BEE was also significantly increased from 0.96 ± 0.12 to 1.00 ± 0.13 after ESD procedure (*p*<0.05, Fig. 3). The stress factor on the postoperative day 1 was computed as 1.06, assuming the stress factor on the day of ESD to be 1.

## Discussion

The physical invasiveness has been expressed by the acceleration of the energy metabolism.^(6–13,24,25)^ It is evident from the significant increases of REE/BW and stress factor of this study that invasiveness in perioperative period of ESD for colorectal cancer enhances the energy metabolism.

In the present study, we calculated both REE/BW and REE/BEE to compare our results with those of previous studies for surgical operation. Although most previous reports have measured the invasiveness of surgical operation with either REE/BW or REE/BEE, they are not the same because formula to calculate BEE is different between male and female. Indeed, in our results, significant increase of REE/BW and REE/BEE was observed in 81.1% and 79.2% of the patients, respectively.

In comparison to the value of the day of colorectal ESD, REE/BW was 6.8% higher on the next day of ESD. Some studies have reported the change of REE in perioperative period of colorectal cancer surgery. In a Western study for gastric and colorectal cancer surgery, REE on postoperative days 7 and 8 was 106.9% of the corresponding preoperative value.^(12)^ A previous Japanese study for surgery of digestive cancers also showed that REE/BW value increased by 18% to 27.0 ± 5.6 kcal/kg/day on the postoperative day 1 as opposed to the preoperative REE/BW value of 22.9 ± 2.5 kcal/kg/day.^(26)^ Both studies indicated that the increase in energy expenditure might be attributed to the invasiveness of surgery for colorectal cancer. In the present study, REE was measured on the postoperative day 1 when the degree of physical and mental burden of the patient is considered to be the highest. The results showed that the increase of REE was only 6.8% and it is considerably lower than that of surgery.

In this study, the stress factor on postoperative day 1 of colorectal ESD was calculated as 1.06. Long *et al.*^(22)^ reported that a stress factor of 1.1 to 1.2 was medium invasion including acute phase in colectomy. In Japan, a previous study reported that surgical colectomy as a medium-degree invasion, which shows a stress factor of 1.4 on the postoperative day 3.^(27)^ The results of present study suggested a significant increase of REE/BEE on postoperative day 1, which means the stress factor of colorectal ESD. However, stress factor remained lower in comparison to that obtained later postoperative days of surgical colon surgery. Recently, surgical techniques, such as laparoscopic surgery, have been developed and widely used for the treatment of colorectal cancer. These treatments might be less-invasive in comparison with open surgery. Further studies are necessary to compare the invasiveness between ESD and less-invasive surgery for colorectal cancer.

Our previous study on gastric ESD showed that energy expenditure per kg of body weight and stress factor also increased during the ESD perioperative period.^(13)^ The study indicated that the stress factor on gastric ESD postoperative day 1 was 1.07. ^(13)^ As described before, colorectal and gastric ESD are different in the perioperative management as well as technical difficulty. However, present study demonstrated that the stress factor on gastric ESD postoperative day 1 was 1.06. These results would indicate that colorectal ESD is a less-invasive procedure which has similar stress factor comparing with gastric ESD.

There are several limitations in this study. First, this study was carried out in a single institute and the number of subjects were relatively small. Indirect calorimeter is a very expensive equipment, and our hospital is only one institution which has indirect calorimeter in our area. As a result, number of subjects with complications of ESD was so small that we could not evaluate the relationship between the energy metabolism and complications of ESD. Second, this is a single arm study without healthy control or patients with surgery. However, it is not possible to perform the same measurement on healthy subjects who underwent non-therapeutic colonoscopy. Further multi-center studies are required to perform accurate comparison of the invasiveness with surgical resection, particularly less-invasive surgery.

In conclusion, the energy expenditure per kg of body weight and stress factor increased during the colorectal ESD perioperative period. However, this increase was low comparing with that experienced for surgery. Furthermore, the invasiveness of colorectal ESD was equal to that of gastric ESD in spite of its technical difficulty. Our findings would contribute to give a better quantitative information about the invasiveness of ESD for the patients who undergo colorectal ESD.

## Figures and Tables

**Table 1:** Basic characteristics of the patients

Parameter	Value
Total patients	53
Age (years old)^a^	71.8 ± 8.4 (49–85)
Sex	
Male:Female	32:21
Multiple lesion’s cases^b^	15 (28.3%)
	(×2: 11, ×3: 4)
Operation time (min)^a^	104.9 ± 66.1 (24–440)
Total resection area (cm^2^)^a^	14.1 ± 14.2 (1.0–98.9)
The maximum length of main 53 lesions (cm)^a^	2.1 ± 1.0 (0.7–7.0)
Complications^b^	
Perforation	1 (1.9%)
Bleeding	2 (3.8%)
Fever (>38.0°C)	1 (1.9%)

^a^Data are presented as mean ± SD (range). ^b^Data are presented as number of positive case (percentage).

**Table 2:** The change of resting energy expenditure (REE), body weight (BW) and basal energy expenditure (BEE) during the perioperative period of colorectal ESD

	The day of ESD	The next day of ESD	

REE (kcal)	1,107.3 ± 204.4	1,139.9 ± 185.2	n.s.

	ESD preoperative state	ESD postoperative state	

BW (kg)	56.5 ± 9.4	54.8 ± 8.9	*p*<0.001
BEE (kcal)	1,075.8 ± 148.5	1,139.9 ± 141.5	*p*<0.001

Data are presented as mean ± SD.
